# The health of rural Black communities during COVID: Some affirmations, some surprises

**DOI:** 10.3389/fpubh.2023.932451

**Published:** 2023-04-13

**Authors:** Sharlene D. Newman, Kimberly Moss, Melonie Pichon, Deborah Scott, Kileema Rogers, Angela Orr, Chuong Bui, Pamela Payne-Foster

**Affiliations:** ^1^Alabama Life Research Institute, The University of Alabama, Tuscaloosa, AL, United States; ^2^Advancing Health Literacy in the Alabama Black Belt, HHS, Fort Deposit, AL, United States; ^3^College of Community Health Sciences, The University of Alabama, Tuscaloosa, AL, United States

**Keywords:** rural, community health, Deep South, African American, mental health

## Abstract

**Background and objective:**

There are overwhelming health disparities in the Deep South. It is important to include the voice of communities affected by these disparities when developing interventions. The goal of the current study was to develop an academic community engaged partnership to strengthen the ability to address priority health concerns of rural African American communities with a focus on health literacy and health advocacy.

**Methods:**

A community-based participatory research approach was used to administer a 15-item community health survey in five rural communities led by African American mayors in Alabama (*N* = 752). The survey assessed the health concerns and the potential behaviors that may be associated with those health concerns.

**Results:**

The five communities demonstrated similarities as well as differences in both the health concerns endorsed and the potential health behaviors that may contribute to those concerns. All five communities identified cardiovascular disease as a health concern with three endorsing mental health issues and 2 dental health. With respect to behaviors, all five communities identified either unhealthy eating/exercise and substance use as concerns with one community identifying racism as a risky behavior affecting health.

**Conclusion:**

The results presented replicate CBPR studies demonstrating that communities are important sources of information about local health priorities and concerns.

## Introduction

The COVID-19 pandemic has highlighted the significant racial health disparities in the U.S. The disparate impact of the pandemic on African Americans was observed across the country but particularly in the Deep South. During the first few months of the pandemic 43% of the deaths in Alabama were African American patients (1) even though they only make up 26% of the population. While poorer health of African Americans and higher rates of chronic disease and diabetes were hypothesized as the explanation for higher rates of COVID among African Americans, disparities in chronic diseases do not explain disparities in COVID deaths (2).

A public health strategy focused on small rural communities with large African American populations is necessary to address racial health disparities in many of the states in the Deep South like Alabama. Alabama is a predominately rural state with 55 of its 67 counties being rural (3). Of those 55 rural counties 10 of them have predominately African American populations, all of which are located in the Alabama Black Belt region. The Alabama Black Belt, named for its rich soil, has a long history of racial injustice and is home to some of the most impoverished communities in the country. Many of the small rural communities in the region have been forgotten. To begin to address the health disparities in these communities, it is important to use a community-based participatory research (CBPR) approach (4, 5) that involves a partnership between academics and communities.

Partnering with rural Black communities using a CBPR approach may be especially critical for a number of reasons. First, the available health data is likely incomplete or unavailable making it important to obtain data from communities. Second, and most importantly, many residents in these communities do not trust researchers [e.g., the U.S. government’s syphilis experiment in Tuskegee (6), a town located in the Alabama Black Belt region]; therefore, trust must be built. CBPR emphasizes equitable engagement with the community and takes a social justice lens including the community in all aspects of the research as decision makers (7).

The current study is the first step in a larger project designed to develop an academic community engaged partnership to strengthen the ability to address priority health concerns of the community. The first step in developing our academic-community partnership was to learn from the communities the health concerns/problems and the behaviors that may contribute to those problems. While there are some commonly reported health disparities associated with Black communities (e.g., diabetes and hypertension) it is important that we not make assumptions about the health or health concerns of a community, as each local community is different and has different needs. The partnership being developed is between the University of Alabama and five rural communities led by African American mayors. We surveyed the five communities using a community health survey developed by the National Association of County and City Health Officials.

## Methods

### The community-academic partnership

The study was conducted by the academic institution, The University of Alabama, Alabama Life Research Institute, and five rural communities. The relationship was initiated with the help of the Alabama Conference of Black mayors. The mayors of five rural communities located across the state of Alabama were interested in improving the health of their communities, particularly in the context of the COVID-19 pandemic. The team was formed to prepare for an application to a funding announcement related to advancing health literacy. The applicant for the funding announcement was required to be communities. The five communities then formed a collaborative and led the effort with the assistance of the academic researchers, placing the community in the leadership role. The characteristics of these communities are provided in Table 1. As shown, these are small, economically disadvantaged communities with high social vulnerability indices (8). While there is no health data specific to each town, the county level data for each community show that they have some of the poorest health outcomes in the state of Alabama, which itself has some of the worse outcomes in the U.S. (9).

**Table 1:** Community characteristics.

Town	Population	% African American	*Per capita* income	SV index
Community 1	1,859	85.5%	$19,618	0.8283
Community 2	2,239	88.4%	$14,872	0.9537
Community 3	1,880	35.4%	$22,793	0.703
Community 4	2,847	64.2%	$10,782	0.9175
Community 5	755	90%	$21,439	0.8729

SV index, social vulnerability index.

### The research team

The study team was composed of researchers, community representatives and mayors. The community representatives were selected by each mayor. These representatives were chosen because they were active in their communities and had knowledge of and access to a diverse range of residents. The team met quarterly in person and biweekly *via* videoconference. During the planning of the project the team decided that it was important to understand the health concerns of each community to guide the development of individualized community intervention plans. It was decided to first administer a community health survey. The five community representatives completed human subjects training and were instructed on verbal consent.

### Participants

All survey participants were adults (*N* = 752; 223 males), aged 18 and older (demographic information is reported in Table 2). The study was approved by the University of Alabama Institutional Review Board. All participants provided verbal consent.

**Table 2:** Demographics of the study participants.

Variable	Categories	Community 1 (*N* = 63/1859)		Community 2 (*N* = 350/2239)		Community 3 (*N* = 71/1880)		Community 4 (*N* = 224/2847)		Community 5 (*N* = 44/755)	
		f	%	f	%	f	%	f	%	f	%
Age	<25	9	15	27	8	19	27	14	6	3	9
	26–29	13	22	53	15	12	17	42	19	8	23
	40–54	22	37	107	31	26	37	87	40	8	23
	55–64	12	20	84	23	5	7	67	30	8	23
	65+	4	7	73	21	9	13	10	5	8	23
Sex	Male	22	35	127	36	22	31	40	18	12	27
Race	Black	41	73	277	81	37	52	120	54	39	100
	Asian							10	4		
	Hispanic			5	1	8	11	7	3		
	Native A					4	7	5	2		
	White	15	27	54	16	22	31	81	36		
	Other			8	2						
Marital	Married	23	37	133	38	39	55	143	64	23	52
Education	<HS	7	12	57	17	15	22	23	10	1	3
	HS grads	42	70	202	59	25	36	132	60	21	53
	College	11	18	79	23	29	42	66	30	17	43
	Other			4	1					1	3
Income	<20 k	8	13	151	46	12	19	102	48	8	20
	20–29 k	21	34	117	36	19	31	110	51	5	13
	30–49 k	26	42	45	14	19	31	22	1	15	38
	50 k+	7	11	15	5	12	19			12	30
Healthcare	Cash	9	15	62	19	24	38	44	20	3	8
	Insurance	38	63	165	49	24	38	93	42	19	48
	Medicaid	6	10	26	8	6	10	28	13	7	18
	Medicare	5	8	65	19	6	10	50	23	5	13
	Veteran	2	3	15	4	3	5	5	2	6	15
	Other			1	0						

f, frequency; %, is the percent of the sample represented; N, sample size/population size. There are some missing data so the sum of frequencies (for each variable) may not be equal to the sample size.

### The survey

A 15-question survey was administered (see Appendix). The survey contained two parts – one assessing community health and another designed to obtain demographic information and was selected by one of the researchers with the approval of the communities. The assessment used is one provided by the Mobilizing for Action through Planning and Partnerships program of the National Association of County and City Health Officials. The survey was designed to identify the community health and quality of life issues within a community. The two questions on the survey that was the primary focus of the current manuscript are: (1) *What do you think are the three most important health problems in our community?* and (2) *What do you think are the three most risky behaviors in our community?* These two questions were the focus because they tell us what the community has identified as the major health problems and the risky behaviors observed in the community, which may be associated with the health problems. Paper and pencil surveys were distributed at community health events organized by the mayors of each community (e.g., covid vaccine clinics, community education evens, and health fairs) as well as *via* canvassing the communities to obtain a representative sample. The number of participants varied across communities. Every effort was made to obtain a diverse sample with respect to age and gender in each town. Community representatives recruited participants at community events, local stores, government offices, and churches. Given the distribution of age and race of the sample, the strategy was successful. However, the larger sample from two towns in particular may give them greater influence on the mean effects. The top five concerns and behaviors for each community were identified by summing the number of times they were selected by participants.

## Results

### The most important “health problems”

The top five ranked health problems each community identified as having the greatest impact on overall community health is presented in Table 3. As shown, cardiovascular disease (e.g., hypertension, heart disease, stroke) was in the top five for all communities with cancer and diabetes being in the top five for 4/5 communities. Mental health and aging issues were in the top five for 3/5 communities with dental problems in 2/5 communities.

**Table 3:** Most endorsed items by community.

	Health problems	Risky behavior
Community 1	Diabetes (61.9%) Heart disease and stroke (49.2%) High blood pressure (42.9) Cancers (38.1%) Dental problems (28.6%)	Lack of exercise (46%) Poor eating habits (42.9%) Being overweight (38.1%) Drug abuse (38.1%) Not getting “shots” to prevent disease (38.1%)
Community 2	Diabetes (49.4%) High blood pressure (45.7%) Cancers (37.4%) Aging problems (32%) Mental health problems (31.4%)	Drug abuse (59.7%) Alcohol abuse (54.6%) Poor eating habits (37.4%) Lack of exercise (34.6%) Being overweight (34.3%)
Community 3	Heart disease and stroke (33.8%) Diabetes (42.2%) Aging problems (33.8%) High blood pressure (26.8%) Dental problems (21.1%)	Alcohol abuse (67.6%) Racism (39.4%) Drug abuse (34.2%) Lack of exercise (26.7%) Poor eating habits (22.5%)
Community 4	Cancers (81.3%) Mental health problems (63.8%) Heart disease and stroke (58.5%) Domestic violence (19.2%) Aging problems (17.9%)	Alcohol abuse (89.7%) Drug abuse (59.4%) Poor eating habits (42.9%) Not getting “shots” to prevent disease (32.1%) Lack of exercise (29%)
Community 5	Mental health problems (40.1%) Diabetes (31.8%) Heart disease and stroke (31.8%) High blood pressure (31.8%) Cancers (25%)	Drug abuse (61.4%) Alcohol abuse (59.1%) Being overweight (34.1%) Lack of exercise (27.3%) Dropping out of school (25%)

The percent of sample who selected the concern/risk is in parentheses.

### The most important “risky behaviors”

Because it is believed that there is a link between behavior/lifestyle and health, assessing the behaviors that the community identified as being associated with health outcomes was also examined. The results revealed some common issues across communities – substance use and unhealthy eating/lack of exercise. In addition, 2/5 communities identified low vaccination rates (likely related to COVID-19 vaccinations), one community identified racism and one low educational attainment as risky behaviors.

### Demographic effects

An odds ratio analysis was performed to assess how demographic variables relate to community health concerns. Demographic variables examined included: race (black, white, others), age (29 or younger, 30–64, 65+), gender, education (<HS, HS, college degree or higher), household income (<29 k, 30–49 k, 50 k+), and whether the participant had private insurance (yes/no). When controlling for community we found that adults 65 years or older were less likely than those 40–64 years to select “mental health problems” as a most important health problem in the community (OR = 0.45, *p* = 0.009). In addition, those who had private insurance were less likely than those without private insurance to select “mental health problems” (OR = 0.62, *p* = 0.012) as a community concern. There were also differences for the selection of “heart disease or stroke.” Adults 65 years or older were more likely than those 29 years or younger to select “heart disease or stroke” (OR = 2.12, *p* = 0.016) as a community health concern. Additionally, adults whose household income was 30–49 k were more likely than those with less than 29 k in income to select “heart disease or stroke” (OR = 1.90, *p* = 0.021). Finally, white adults were less likely than Black adults to select “diabetes” (OR = 0.59, *p* = 0.031) as a concern.

When examining behaviors that impact community health, adults of other race were less likely than Black adults to select “alcohol abuse” as a most important “risky behavior” in the community (OR = 0.42, *p* = 0.027). Adults 65 years or older were also less likely than those 40–64 years old to select “alcohol abuse” (OR = 0.49, *p* = 0.010) as a “risky behavior.” Age also was associated with the identification of “lack of exercise” as a “risky behavior” with adults 65 years or older being more likely than those 64 years or younger to select “lack of exercise” (65+ vs. 40–64, OR = 2.17, *p* = 0.004; 65+ vs. 29 or less, OR = 1.99, *p* = 0.017).

## Discussion

The current study was the first step in a plan to address priority health concerns in rural African American communities with a focus on health literacy and health advocacy. This first step was designed to learn about the health concerns as well as the behaviors that the community associate with those health concerns using a CBPR approach. The information learned will be used to design follow-up assessments and individualized interventions.

The five communities identified health concerns that have been shown to be a problem in rural Alabama and that have been found to have significant racial disparities – diabetes and cardiovascular disease (i.e., high blood pressure and stroke). For example, Alabama has a higher rate of diabetes (14.8%) than the national average (10.6%) (2). The same is true for hypertension with Alabamians having a 10-percentage point higher rate than the national average (10). The stroke mortality rate in Alabama is the second highest in the US (11). For all three health conditions, racial disparities have been found with African American residents having higher rates of diabetes, high blood pressure and stroke than White residents. Therefore, finding these health conditions as primary concerns for the residents is a validity check on the surveys and is in line with the many CBPR studies that have demonstrated that communities are very capable of identifying commonly reported health concerns (12, 13). While the survey did not ask participants to directly link behaviors to health outcomes, the unhealthy eating and exercise habits identified can easily be associated with the diabetes and cardiovascular concerns. Interventions developed in collaboration with the communities to address these risk factors along with culturally appropriate education about these chronic health conditions may be effective in reducing the rural and racial disparities observed. It should be noted that addressing these risk factors also includes addressing systemic issues including lack of access to quality healthcare and foods. For example, many low-income communities, including rural communities, are in food deserts making it difficult to manage diets and therefore health. Many rural African American Alabama communities do not have grocery stores and unlike rural communities in other parts of the country, these are no farming communities so there is a lack of access to fresh produce. In 2018, Feeding America estimated the rate of food insecurity in Alabama was 23% (14) with numbers increasing during the pandemic.

Cancer is also a concern in four out of the five communities. In Alabama, it is estimated that 10,530 people will die from cancer a year (15). African Americans living in rural areas are at higher risk of dying from cancers (16). They are often diagnosed with advanced disease (17), have poor access to timely and appropriate medical care (18), and have lower survival rates even when diagnosed with early-stage disease. The root causes of these disparities are not clearly described or documented. What is abundantly clear is that health inequities are lingering and deep-rooted. In addition, AL has more than its share of landfills (19) which are primarily placed in low-income African American communities (20). Cancer risk has been shown to be connected to living near these sites (21, 22), which may also increase the communities’ concerns.

Mental health was a top ranked concern for three of the five communities. Although the literature suggests that African Americans, particularly in rural areas are unwilling to discuss mental health due to stigma (23, 24), the survey participants rate it as a major concern. This suggests that even if they are unwilling to address the problem openly, they are willing to acknowledge that it is a problem. Stigma is likely a barrier to help-seeking (25, 26). However, given its high ranking there may be an opportunity for de-stigmatization interventions to be effective to allow for increased help seeking and more open conversations about mental health. It should be noted that recently young African American adults have shown an increase in conversations about mental health and self-care (27, 28), providing an opportunity to engage in important conversations with the youth in these communities. This trend can also be seen in the data presented in that older adults were less likely to report that mental health was a problem in the community. The mental health concern in the surveyed communities is likely linked to *substance use* being identified as a top-rated concerning behavior. Because of the high poverty rates and high social vulnerability indices, many of the individuals living in the five communities surveyed are experiencing significant economic stress likely affecting housing, food and health care access. Finding that individuals who do not have private insurance were more likely to report that mental health was a community concern supports this hypothesis. This chronic stress may be expected to result in poor health, including increases in depression and anxiety, as well as poor coping strategies, including substance use and unhealthy eating habits. Interventions designed to address the chronic stress residents are experiencing, training on more effective coping strategies, along with de-stigmatizing mental health and substance use disorders may be critical to addressing the concerns expressed in the survey.

Finally, aging related concerns were identified in two of the five communities. Access to care is a problem in rural areas with access to specialty care almost non-existent. This includes geriatric specialists. While the survey did not allow for specific information about aging related concerns, some may include hearing loss, mobility issues including fall risk, and cognitive decline. There are few assisted living communities and public transportation is not available in rural areas which contributes to the problem. A more in-depth assessment of the aging concerns is necessary to fully characterize the issue.

Another interesting finding is that when asked to select behaviors that have the greatest impact on community health, one community identified racism. The community that selected racism as a top five risky behavior is the community with the smallest percentage of African American residents. In fact, it is the only community surveyed in which the African American population is in the minority. Further investigation is necessary to understand why racism was selected as well as what is meant when racism is referred to as a risky behavior. However, it does suggest that the residents are experiencing more interpersonal racism that may affect their access to healthcare.

## Conclusion and implications

The study findings have important implications for future research as well as public health. First, it reinforces the findings from previous CBPR studies showing that the community is a reliable source of information related to the health concerns and behaviors of its residents (12, 13). The survey results identified the major health concerns that are easily identified in health data (e.g., diabetes and high blood pressure) as well as concerns that are more community specific which have not been well documented. There is a wealth of research that demonstrates the importance of CBPR and community involvement in developing and implementing public health interventions (29). In addition to identifying health concerns, the communities also identified risky behaviors, with some of them were unexpected and may provide important new directions for research (e.g., racism). The second implication, and somewhat surprising finding, is that the rural African American communities in Alabama are concerned about mental health, identifying it as a major health concern and substance use as a major risk factor. This is important because it demonstrates that the community attitudes may be shifting which affects the interpretation of research results as well as the development and deployment of interventions. For example, stigma associated with mental health may still be a barrier but the fact that the majority of respondents listed it is a concern suggests that the stigma may be declining. It is important to collaborate with individual communities to develop interventions specific to their community. Therefore, understanding the source of the stigma, and providing educational resources designed in collaboration with the community will be essential. It will also important to address the chronic stress experienced by communities by providing culturally appropriate education about healthy coping strategies and the links between stress and the development of chronic disease, and substance use and psychological disorders. This may be even more pronounced after COVID and should be explored further. Finally, the underlying causes of increased stress in these communities should also be addressed. The poverty is systemic and the result of decades of neglect and in many cases systemic racism. Without addressing these root causes of health disparities, it will be impossible to eliminate them.

## Data availability statement

The raw data supporting the conclusions of this article will be made available by the authors, without undue reservation.

## Ethics statement

The studies involving human participants were reviewed and approved by the University of Alabama. Written informed consent for participation was not required for this study in accordance with the national legislation and the institutional requirements.

## Author contributions

SN performed the material preparation and analysis. KM, MP, DS, KR, and AO performed the data collection. SN and PP-F wrote the first draft of the manuscript. CB performed statistical analyses. All authors commented on previous versions of the manuscript, contributed to the study, read, and approved the final manuscript.

## Funding

This work was supported by a grant from HHS (# CPIMP211265) awarded to the Town of Fort Deposit.

## Conflict of interest

The authors declare that the research was conducted in the absence of any commercial or financial relationships that could be construed as a potential conflict of interest.

## Publisher’s note

All claims expressed in this article are solely those of the authors and do not necessarily represent those of their affiliated organizations, or those of the publisher, the editors and the reviewers. Any product that may be evaluated in this article, or claim that may be made by its manufacturer, is not guaranteed or endorsed by the publisher.
