# Supplementary material for: The health of rural Black communities during COVID: Some affirmations, some surprises
Source: Front Public Health. 2023 Apr 13;11:932451. doi: 10.3389/fpubh.2023.932451 (PMC10133505; doi:10.3389/fpubh.2023.932451)
Supplement: Supplementary file 1 [file Data_Sheet_1.pdf]

Supplementary Materials

Community Health Survey

1. In the following list, what do you think are **the three most important factors for a "Healthy Community?"** (Those factors which most improve the quality of life in a community.)

Check only three:

___ Good place to raise children
___ Low crime / safe neighborhoods
___ Low level of child abuse
___ Good schools
___ Access to health care (e.g., family doctor)
___ Parks and recreation
___ Clean environment
___ Affordable housing
___ Arts and cultural events
___ Excellent race relations
___ Good jobs and healthy economy
___ Strong family life
___ Healthy behaviors and lifestyles
___ Low adult death and disease rates
___ Low infant deaths
___ Religious or spiritual values
___ Other______________________________

2. In the following list, what do you think are **the three most important "health problems"** in our community? (Those problems which have the greatest impact on overall community health.)

Check only three:

___ Aging problems (e.g., arthritis, hearing/vision loss, etc.)
___ Cancers
___ Child abuse / neglect
___ Dental problems
___ Diabetes
___ Domestic Violence
___ Firearm-related injuries
___ Heart disease and stroke
___ High blood pressure
___ HIV / AIDS
___ Homicide
___ Infant Death
___ Infectious Diseases (e.g., hepatitis, TB, etc.)
___ Mental health problems
___ Motor vehicle crash injuries
___ Rape / sexual assault
___ Respiratory / lung disease
___ Sexually Transmitted Diseases (STDs)
___ Suicide
___ Teenage pregnancy
___ Other _____________________

3. In the following list, what do you think are **the three most important "risky behaviors"** in our community? (Those behaviors which have the greatest impact on overall community health.)

Check only three:

___ Alcohol abuse
___ Being overweight
___ Dropping out of school
___ Drug abuse
___ Lack of exercise
___ Poor eating habits
___ Not getting "shots" to prevent disease
___ Racism
___ Tobacco use
___ Not using birth control
___ Not using seat belts / child safety seats
___ Unsafe sex
___ Other______________________________

4. How would rate our community as a "Healthy Community?"

___ Very unhealthy ___ Unhealthy ___ Somewhat healthy ___ Healthy ___ Very healthy

5. How would rate your own personal health?

___ Very unhealthy ___ Unhealthy ___ Somewhat healthy ___ Healthy ___ Very healthy

6. Approximately how many hours per month do you volunteer your time to community service? (e.g., schools, voluntary organizations, churches, hospitals, etc.)

___ None ___ 1 - 5 hours ___ 6 - 10 hours ___ Over 10 hours

Please answer questions #7-15 so we can see how different types of people feel about local health issues.

7. Zip code where you live: ___________

8. Age:
___ 25 or less
___ 26 - 39
___ 40 - 54
___ 55 - 64
___ 65 or over

9. Sex: ___ Male ___ Female

10. Ethnic group you most identify with:
___ African American / Black
___ Asian / Pacific Islander
___ Hispanic / Latino
___ Native American
___ White / Caucasian
___ Other _________________

11. Marital Status:
___ Married / co-habitating
___ Not married / Single

12. Education
___ Less than high school
___ High school diploma or GED
___ College degree or higher
___ Other__________________

13. Household income
___ Less than $20,000
___ $20,000 to $29,999
___ $30,000 to $49,999
___ Over $50,000

14. How do you pay for your health care? (check all that apply)
___ Pay cash (no insurance)
___ Health insurance (e.g., private insurance, Blue Shield, HMO)
___ Medicaid
___ Medicare
___ Veterans' Administration
___ Indian Health Services
___ Other ____________________

15. Where / how you got this survey: (check one)
___ Church
___ Community Meeting
___ Grocery Store / Shopping Mall
___ Mail
___ Newspaper
___ Newsletter
___ Personal Contact
___ Workplace
___ Other ____________________
